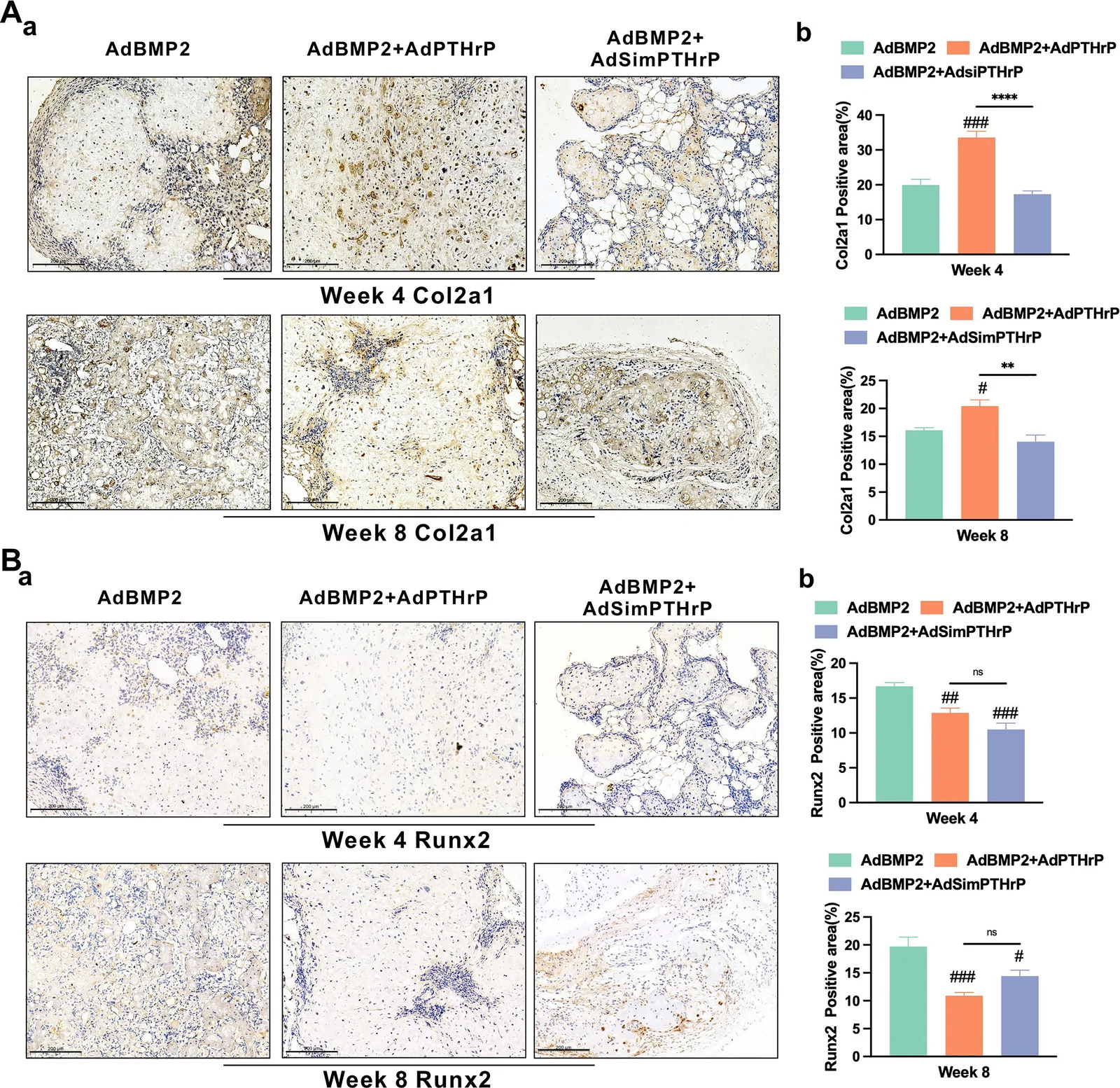# PTHrP suppresses BMP2-induced osteochondral differentiation and preserves cartilage phenotype in mesenchymal stem cells via inactivating PI3K–AKT signaling

**DOI:** 10.1016/j.gendis.2026.102273

**Published:** 2026-06-05

**Authors:** Shengqiang Gao, Piao Zhao, Tingting Du, Yu Ye, Shengwen Cheng, Senrui Liu, Chengcheng Du, Tong-Chuan He, Wei Huang, Zhenglin Zhu, Wei Xu, Junyi Liao

**Affiliations:** a Department of Orthopaedic Surgery, Chongqing Municipal Health Commission Key Laboratory of Musculoskeletal Regeneration and Translational Medicine, Orthopaedic Research Laboratory, The First Affiliated Hospital of Chongqing Medical University, Chongqing 400016, China; b Molecular Oncology Laboratory, Department of Orthopaedic Surgery and Rehabilitation Medicine, The University of Chicago, Medical Center, Chicago, IL 60637, USA; c Medical Examination Center, The First Affiliated Hospital of Chongqing Medical University, Chongqing 400016, China; d Department of Orthopaedic Surgery, The Second People’s Hospital of Chongqing Yubei District, Chongqing 401147, China

**Keywords:** BMP2, Cartilage tissue engineering, Chondrogenesis, Endochondral ossification, Hypertrophic chondrocytes, MSCs, PI3K–AKT signaling, PTHrP

## Abstract

Bone morphogenetic protein 2 (BMP2) has the potential to drive multipotent mesenchymal stem cells (MSCs) to undergo chondral–osteogenic (including hypertrophic chondrocyte) differentiation. Parathyroid hormone-related protein (PTHrP) plays an essential role in fetal endochondral ossification, although persistent PTHrP stimulation leads to postnatal bone loss. Here, we investigated the effects of PTHrP on BMP2-induced endochondral ossification through hypertrophic chondrocyte differentiation of MSCs. We found that silencing PTHrP expression inhibited BMP2-induced chondrogenic, hypertrophic, and osteogenic differentiation of MSCs, while overexpression of PTHrP promoted BMP2-induced chondrogenic differentiation of MSCs and suppressed BMP2-induced hypertrophic differentiation of MSCs. Using stem cell implantation studies in athymic nude mice, we demonstrated that while MSCs with silenced PTHrP expression exhibited significantly reduced endochondral ossification of subcutaneous cartilage, overexpression of PTHrP promoted the growth of subcutaneous cartilage while significantly delaying ossification, leading to the preservation of cartilage phenotype. Mechanistically, we showed that exogenous PTHrP promoted BMP2-induced chondrogenic differentiation of MSCs by up-regulating Sox9 expression and inactivating PI3K–AKT signaling. Collectively, our findings strongly suggest that PTHrP may play an important role in mediating BMP2-induced chondral-osteogenic differentiation of MSCs by inactivating PI3K–AKT signaling, indicating that PTHrP may be exploited for endochondral cartilage-bone tissue engineering.

## Introduction

Stem cell-derived and growth factor-enhanced cartilage regeneration is meaningful for dealing with cartilaginous defects, like traumatic defects.^1^, ^2^, ^3^, ^4^ As multipotent precursors of connective tissue cells, mesenchymal stem cells (MSCs) have the potential to multilineage differentiation, including chondrogenic differentiation. On account of the ease of isolation, stable expression of exogenous genes, and abundance of source, MSCs were recognized as perfect seed cells for regenerative medicine.^1^^,^^2^^,^^5^, ^6^, ^7^ As MSC-based tissue engineering cartilage was identified as an ideal substitute for cartilage defect,^8^^,^^9^ directing MSCs towards chondrogenic differentiation is fundamental for constructing tissue-engineered cartilage. MSC-mediated cartilage regeneration is based on the chondrogenic differentiation and chondrocyte phenotype maintenance, which signifies that hypertrophic and osteogenic differentiation are impeding cartilage regeneration.^10^^,^^11^

Belonging to the transforming growth factor beta (TGF-β) super-family, bone morphogenetic protein 2 (BMP2) is featured as effectively guiding MSC chondrogenic differentiation.^12^^,^^13^ In addition to chondrogenic differentiation, BMP2 is also characterized to drive osteogenic differentiation as well as hypertrophic differentiation afterwards in MSCs. These phenomena directly contradict BMP2’s ability to maintain cartilage phenotypes.^14^, ^15^, ^16^, ^17^ We previously clarified that BMP2 mediated chondrogenic differentiation of stem cells by up-regulating the expression of key transcription factor, SRY-box transcription factor 9 (Sox9), and overexpression of Sox9 promoted BMP2-triggered chondrogenic differentiation and restrained BMP2-induced osteogenic differentiation of stem cells.^14^^,^^15^ However, BMP2-stimulated hypertrophic differentiation after chondrogenic differentiation still limited the further application of BMP2-based cartilage engineering.^14^^,^^15^^,^^18^ Hence, clarifying the mechanisms involved in BMP2-induced chondrogenic differentiation, especially following hypertrophic chondrogenic differentiation, is essential for the application of BMP2-mediated tissue engineering.

Parathyroid hormone-related protein (PTHrP) is known to play an essential role in fetal endochondral ossification, where bone is generated within a cartilage primordium.^19^ As a versatile protein hormone, PTHrP is viewed as having autocrine or paracrine functions in the early mesenchyme and immature cartilage of the vertebral column, tail, and long bones.^20^^,^^21^ With the onset of ossification, expression of PTHrP gradually decreased, and it was only expressed in chondrocytes and osteoblasts. During endochondral ossification, Indian hedgehog (Ihh) expressed by hypertrophic chondrocytes and osteoblasts prompted the expression of PTHrP in pre-hypertrophic and hypertrophic chondrocytes, simultaneously facilitating the PTHrP secretion in proliferating chondrocytes, promoting the proliferation of chondrocytes, and inhibiting the hypertrophic differentiation.^19^, ^20^, ^21^, ^22^ At the same time, the increased PTHrP attenuated the expression of Ihh.^19^ In other words, the feedback of Ihh–PTHrP regulates the endochondral ossification during the development of long bones. While studies identified the essential role of PTHrP in bone formation,^23^^,^^24^ continuous infusion of PTHrP suppresses bone formation and uncouples bone turnover,^25^ indicating that PTHrP alone may exert a two-phased effect on bone formation. Therefore, it is critical to delineate the regulatory roles of PTHrP at the different stages of MSC differentiation in the presence of other chondrogenic/osteogenic factors such as BMP2.

In this study, by conducting *in vitro* and *in vivo* studies, we sought to investigate the impact of PTHrP on BMP2-induced chondral–osteogenic differentiation through hypertrophic differentiation of MSCs. We found that through RNAi silencing studies, PTHrP was essential for BMP2-induced chondral–osteogenic and hypertrophic differentiation of MSCs, whereas exogenous overexpression of PTHrP potentiated BMP2-regulated chondrogenic differentiation of MSCs while maintaining cartilage phenotype. Mechanistically, PTHrP was shown to potentiate BMP2-induced hypertrophic–chondrocyte differentiation of MSCs by inactivating phosphatidylinositol 3-kinase (PI3K)–protein kinase B (AKT) signaling, suggesting a regulatory role of PTHrP in BMP2-initiated chondrogenic differentiation. Thus, PTHrP may be further exploited to augment BMP2-based cartilage and bone tissue engineering.

## Materials and methods

### Ethics statement and animal care and use

The animal protocol of this study was reviewed and approved by the Ethical Committee of the First Affiliated Hospital of Chongqing Medical University (No. 2022-K92). Animal surgery was done under anesthesia according to the approved animal protocol, and kept in barrier cages with standard conditions. Mice were euthanized under carbon dioxide (CO_2_) anesthesia at the indicated time points. All necessary efforts were made to reduce the animals’ suffering, and ectopic masses were collected after confirming that nude mice had respiratory and cardiac arrest and dilated pupils.

### Cell culture medium and chemicals

Human embryonic kidney cell line HEK 293 and mouse mesenchymal stem cell line C3H10T1/2 were obtained from American Type Culture Collection (ATCC, Manassas, Virginia, USA). Cells were maintained in complete Dulbecco’s modified Eagle’s medium (DMEM, Hyclone, catalog: SH30243.FS, China) supplemented containing 10% fetal bovine serum (Gibco, catalog:12664025C, Australia), 100 U/mL penicillin, and 100 mg/mL streptomycin at 37 °C in humidified 5% CO_2_. Unless indicated otherwise, all chemicals were purchased from Thermo-Fisher Scientific or Sigma–Aldrich.

### Construction and generation of recombinant adenoviral vectors AdBMP2, AdPTHrP, AdsimPTHrP, AdGFP, and AdRFP

Recombinant adenoviruses were constructed using the AdEasy technology.^26^^,^^27^ The coding regions of human BMP2 and mouse PTHrP were amplified by high-fidelity PCR and subcloned into an adenoviral shuttle vector to generate recombinant adenoviral vectors. HEK 293 cells were used to generate recombinant adenoviruses by transfecting them with the recombinant adenoviral vectors. Similarly, an adenoviral shuttle vector pAd-Trace-OK-simPTHrP19 was made by assembling three small interfering RNAs (siRNAs) targeting mouse PTHrP simultaneously using the Gibson Assembly system.^28^ The resulting adenoviruses were designated as AdGFP (green fluorescent protein, GFP), AdRFP (red fluorescent protein, RFP), AdBMP2, AdPTHrP, and AdsimPTHrP. The AdGFP and AdRFP were used as mock vector controls.

### Micromass chondrogenic differentiation of MSCs *in vitro*

Micromass culture was used to mimic mesenchyme condensation and induce the differentiation of MSCs into chondrogenesis. C3H10T1/2 cells infected with the indicated adenovirus vectors were harvested when they grew to 80%–90% confluency, and subsequently resuspended at the center of each well in the 12-well plates in high density (approximately 10^5^ per 25 μL medium) to form a micromass of about 5 mm in diameter, and then incubated in a CO_2_ incubator. Complete DMEM (3 mL ) was added to each well at 2 h after incubation, and 50% of the culture medium was replaced with fresh medium every two days.

### Alcian blue staining assay

Alcian blue staining was performed as previously described for micromass pellets.^14^^,^^27^^,^^29^ At indicated time points, medium was removed, pellets were washed in phosphate-buffered saline, fixed in 4% paraformaldehyde, and then washed again in phosphate-buffered saline, followed by staining with Alcian blue (0.5%) in 0.1 M HCl (pH 1.0) for 12 h and then washing with distilled water. The alcian blue-stained pellets were dissolved in 6 M guanidine hydrochloride at room temperature, and the optical absorbance was measured by a microplate reader (xMark, Bio-Rad, USA) at 630 nm.

### Alkaline phosphatase (ALP) assay

Both quantitative and qualitative assessments of ALP activities were conducted as described previously.^30^^,^^31^ Briefly, treated cells were fixed with 0.05% glutaraldehyde at room temperature for 10 min and stained with 0.1 mg/mL of napthol AS-MX phosphate mixed with 0.6 mg/mL of Fast Blue BB salt at room temperature for 20 min. For quantitative assays, treated cells were lysed with Cell Culture Lysis Buffer (Promega, catalog: E1531, Madison, Wisconsin, USA) and incubated with chemiluminescence substrate and Lupo buffer (BD Clontech). All assays were performed in triplicate, and ALP activities were normalized by total cellular protein concentrations.

### RNA isolation and quantitative reverse transcription PCR

The total RNA extraction kit (AG catalog: AG21023, Hunan, China) was used for total RNA extraction. Total RNA was subjected to reverse transcription reactions using PrimeScript RT reagent kit (MCE catalog: HY-K0511A, USA). The quantitative PCR analysis was done with the use of CFX96 Real-Time PCR Detection System (Bio-Rad, California, USA) with MCE SYBR Green qPCR Master Mix (MCE catalog: HY-K0523, USA). PCR procedures were strictly followed: 95 °C for 5 min, and then 40 cycles of 95 °C for 10 s, 60 °C for 20 s, and 72 °C for 20 s. Quantitative reverse transcription PCR results were analyzed by the Bio-Rad CFX Manager software. Since the adenovirus tool was constructed using human BMP2, the BMP2 primers used for quantitative PCR detection were human sequences, while the primers for other markers were mouse sequences homologous to C3H10T1/2. The PCR primer sequences are listed in Table S1. β-Actin was used as the internal control of mRNA.

### Western blotting analysis

Total cellular lysates were prepared by sonication in radioimmunoprecipitation assay (RIPA) buffer (Beyotime Biotechnology, catalog: P0038, China) containing 1% phosphatase inhibitors (Bimake, catalog: B14001, China) and 1% protease inhibitors (Bimake, catalog: B14001, China), followed by centrifugation to remove cell debris. The supernatant was boiled, and total protein concentrations were determined using the bicinchoninic acid assay kit (Beyotime Biotechnology, catalog: P0010, China). Equal amounts of proteins were electrophoresed on 7%–12.5% SDS-PAGE gels (Omni-Easy™ One-Step PAGE Gel Fast Preparation Kit, catalog: PG210, EpiZyme, China) and then transferred to PVDF membrane (0.45 μm, Millipore, catalog: IPVH00010, Massachusetts, USA). Membranes were blocked with 5% skimmed milk at room temperature for 1 h and incubated at 4 °C overnight with the following primary antibodies: BMP2 (ImmunoWay, catalog: YM8215, USA, 1:1000), Sox9 (Zen Bio, catalog: R380995, China, 1:2000), COL2a1 (Zen Bio, catalog: 222189, China, 1:1000), COL10a1 (Santa Cruz Biotechnology, catalog: sc-59954, Texas, USA, 1:1000), Runx2 (Abcam, catalog: ab76956, Cambridge, USA, 1:1000), PTHrP (ImmunoWay, catalog: YT5790, USA, 1:1000), AKT (Abcam, catalog: ab179463, Cambridge, USA, 1:1000), phosphor-AKT (Abcam, catalog: ab192623, Cambridge, USA, 1:1000), PI3kP85, (Abcam, catalog: ab191606, Cambridge, USA, 1:1000), and GAPDH (Zen Bio, catalog: R380626, 1:2000). After being washed, the membranes were incubated with corresponding secondary antibodies conjugated with horseradish peroxidase (HRP, Zen Bio, catalog: 18008, China, 1:2000). The presence of interested proteins was visualized with Immobilon Western Chemiluminescent HRP Substrate (Abcam, Cambridge, catalog: ab5801, USA). Relative protein levels were calculated using ImageJ software.

### Stem cell subcutaneous implantation

The experimental procedures involving animal use and care were approved by the Chongqing Medical University Animal Care and Use Committee. The subcutaneous stem cell implantation procedures were carried out as previously described.^14^^,^^29^^,^^30^^,^^32^^,^^33^ Briefly, MSCs were infected with AdBMP2, AdGFP, AdRFP, AdPTHrP, or AdsimPTHrP. The cells were collected at 48 h after infection, mixed in DMEM-diluted Matrigel (Corning, catalog: 354230, New York, USA), and injected subcutaneously into athymic nude mice flanks (4 weeks old, female, *n* = 3/group, 4 × 10^6^ cells per injection). Mice were euthanized to retrieve ectopic masses at 4 and 8 weeks after implantation, respectively. Neither the AdGFP nor the AdRFP control groups developed subcutaneous masses at any tested time points.

### Micro-CT analysis

Bone formation of the retrieved masses was assessed with a Micro-CT imaging system (VivaCT80, CANCO Medical AG, Switzerland). Scanning parameters were 30 min/sample, 10 mm spacing between layers, 70 KV scanning voltage, and 114 mA current. The three-dimensional isosurfaces of bony masses were reconstructed and analyzed using Scanco image analysis software.

### Hematoxylin–eosin staining and safranin-O-fast blue staining

Deparaffinized sections were rehydrated and stained with hematoxylin and eosin or Safranin-O-fast blue according to our standard protocols.^7^^,^^34^^,^^35^ Staining results were recorded using a microscope (IX73, Olympus, Japan) for histological assessment.

### Immunohistochemistry assay

Sections were deparaffinized and rehydrated, treated with 3% hydrogen peroxide for 10 min, boiled in citrate buffer (pH 6.0) at 95–100 °C for 20 min, and blocked with goat serum. The blocked sections were incubated with primary antibody against Col2a1 (Abcam, catalog: ab307674, Cambridge, USA, 1:200 dilution) or Runx2 (Cell Signaling Technology, catalog: 12556, Massachusetts, USA, 1:200 dilution) at 4 °C overnight. Subsequently, the sections were washed and incubated with respective biotin-labeled secondary antibodies for 30 min, and then incubated at room temperature with streptavidin-HRP conjugate for 20 min. Negative control was set up with staining without a primary antibody. Images were documented under a bright-field microscope (IX73, Olympus, Japan).

### Statistical analysis

Unless stated otherwise, all quantitative tests were done in biological or technical triplicate. Quantitative data were expressed as mean ± standard deviation and analyzed with GraphPad Prism software (Version 9.3.1, GraphPad Software, USA). The Shapiro–Wilk test was used for the normality test of the data. Student’s *t*-test and one-way analysis of variance (ANOVA) were used for statistical analysis. *P* < 0.05 was considered statistically significant.

## Results

### Silencing endogenous PTHrP expression attenuates BMP2-induced chondrogenic, hypertrophic, and osteogenic differentiation of MSCs

To effectively overexpress or silence target genes, we engineered adenoviral vectors to express BMP2, PTHrP, or siRNAs that silence PTHrP expression, while adenoviral vectors expressing GFP or RFP only were used as mock virus controls. We demonstrated that these adenoviral vectors infected the MSCs with high efficiency alone or in combination (data not shown; Fig. S1A). As expected, AdBMP2 and AdPTHrP were shown to effectively express BMP2 and PTHrP at mRNA (Figs. S1B, a, b) and protein (Fig. S1C, a–c; Fig. 1D, a, b). Conversely, AdsimPTHrP was shown to effectively down-regulate PTHrP expression at both mRNA and protein levels (Fig. S1B and c; Fig. 1D, c).

**Figure 1:**
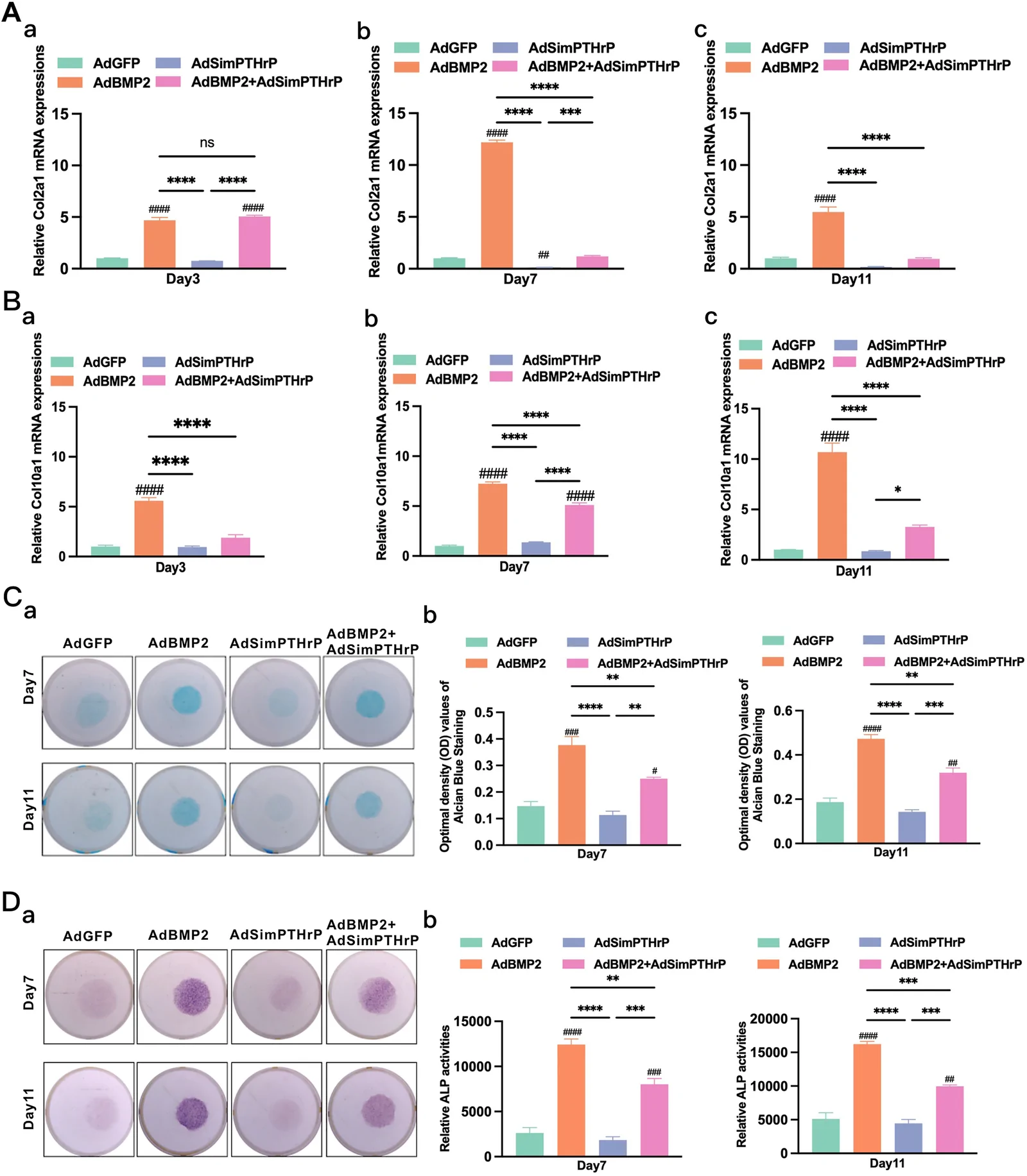
Silencing PTHrP expression attenuated BMP2-induced chondrogenic, hypertrophic, and osteogenic differentiation of mesenchymal stem cells. **(A)** Silencing PTHrP inhibited BMP2-induced Col2a1 gene expression. BMP2 up-regulated Col2a1 gene expression on days 3 (a), 7 (b), and 11 (c), while silencing PTHrP significantly inhibited BMP2-induced Col2a1 expression on days 7 and 11. **(B)** Silencing PTHrP inhibited BMP2-induced Col10a1 gene expression. BMP2 dramatically up-regulated Col10a1 gene expression on days 3 (a), 7 (b), and 11 (c), while silencing PTHrP significantly inhibited BMP2-induced Col10a1 expression on days 3, 7, and 11. **(C)** Silencing PTHrP inhibited BMP2-induced aggrecan expression. (a) Alcian blue staining was used to detect Aggrecan production on days 7 and 11. (b) Quantitative analysis also showed that silencing PTHrP significantly inhibited BMP2-induced Aggrecan synthesis on days 7 and 11. **(D)** Silencing PTHrP inhibited BMP2-induced ALP activity. ALP staining (a) and ALP reading (b) were used to assess ALP activities. Morphological staining on days 7 and 11 was shown (a), and quantitative analysis also showed that silencing PTHrP significantly inhibited BMP2-induced ALP activities on days 7 and 11 (b). ^##^*p* < 0.01, ^###^*p* < 0.001, and ^####^*p* < 0.0001, compared with the control group. ∗*p* < 0.5, ∗∗*p* < 0.01, ∗∗∗*p* < 0.001, and ∗∗∗∗*p* < 0.0001, compared with the indicated group.

Since BMP2 is known to induce both chondrogenic and osteogenic differentiation, we first determined the effects of silencing PTHrP expression on BMP2-induced osteogenic and chondrogenic differentiation of MSCs. Col2a1 is the marker of chondrogenic differentiation and is up-regulated at the early stage of chondrogenesis. We found that BMP2 effectively induced Col2a1 expression from day 3 to day 11, peaking at day 7. However, silencing PTHrP led to decreased expression of BMP2-induced Col2a1 on day 7 and day 11 (Fig. 1A). Furthermore, BMP2-induced expression of hypertrophic differentiation marker Col10a1 from day 3 to day 11 was significantly suppressed when endogenous PTHrP was silenced (Fig. 1B). Alcian blue staining and quantitative analysis showed that BMP2 induced aggrecan synthesis that could be inhibited by silencing PTHrP (Fig. 1C, a, b). ALP staining and quantitative assays also showed that BMP2-induced ALP activities were suppressed by silencing PTHrP (Fig. 1D, a, b). These results suggest that PTHrP may serve as an essential mediator for BMP2-induced chondrogenic and hypertrophic differentiation of MSCs.

### Exogenous PTHrP inhibits BMP2-induced hypertrophic–chondrogenic differentiation of MSCs while promoting cartilage phenotype

We next investigated the effects of exogenous PTHrP on BMP2-induced osteogenic and endochondrogenic differentiation of MSCs. Adenovirus-mediated overexpression of PTHrP was shown to inhibit BMP2-induced Col2a1 expression on day 3 and day 7 (but less pronounced at day 11) when compared with the AdBMP2 alone group (Fig. 2A, a, b). The hypertrophic differentiation marker Col10a1 was effectively induced by BMP2, which was inhibited by AdPTHrP expression at day 3 and day 11 (Fig. 2B, a, b). However, Alcian blue staining of micromass assays indicated that BMP2 induced aggrecan synthesis, which was potentiated by AdPTHrP on day 7, although no statistically significant difference was observed on day 11 (Fig. 2C, a, b). Interestingly, ALP assays showed that overexpression of PTHrP did not influence BMP2-induced osteogenic differentiation of MSCs in *in vitro* micromass assays (Fig. 2D, a, b). Collectively, these results suggest that PTHrP may play an important biphasic role in maintaining chondrocyte phenotype in BMP2-induced hypertrophic–chondrosteogenic differentiation of MSCs.

**Figure 2:**
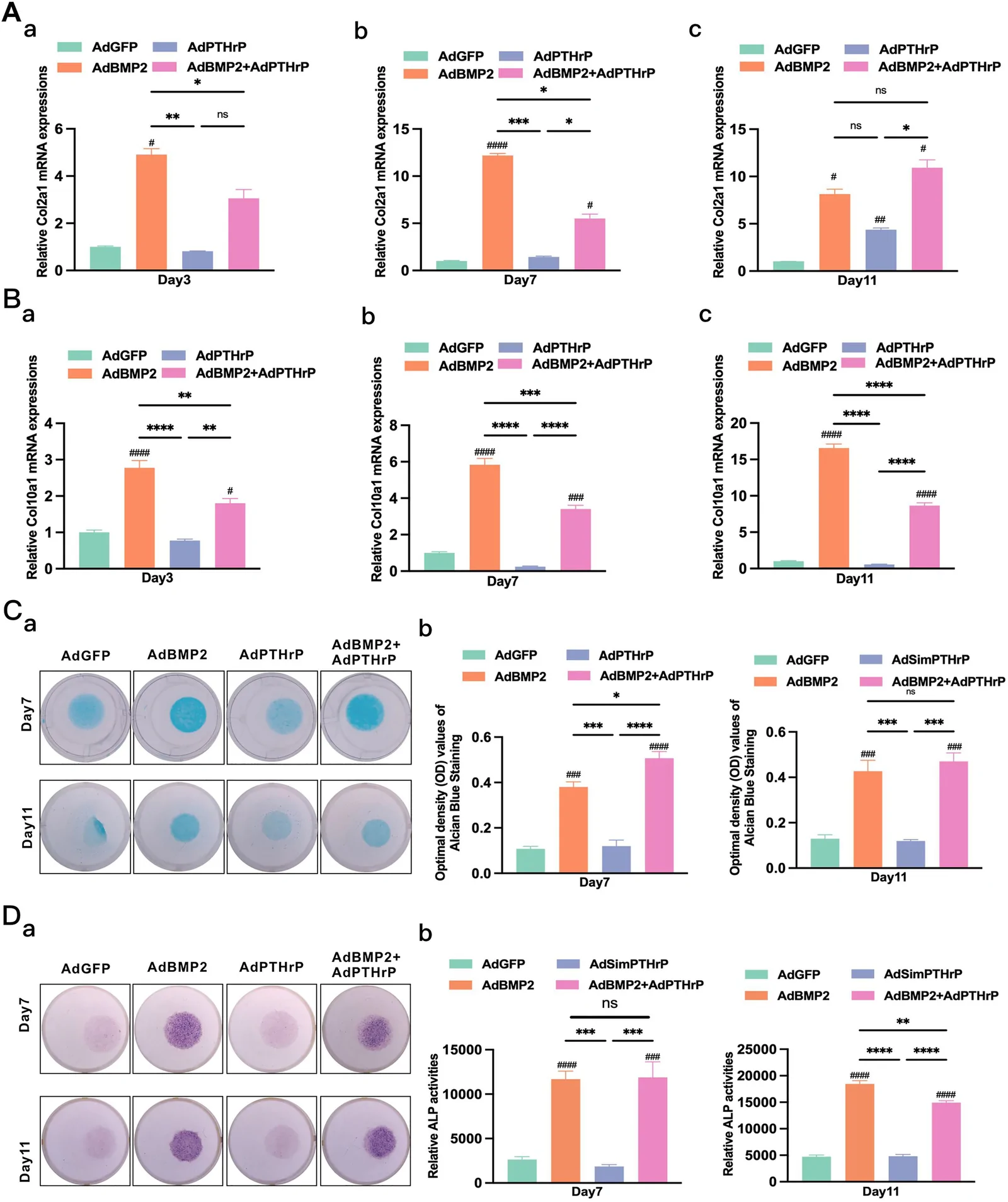
PTHrP overexpression inhibits BMP2-induced hypertrophic and osteogenic differentiation of mesenchymal stem cells. **(A)** Overexpression of PTHrP inhibited BMP2-induced Col2a1 gene expression. BMP2 up-regulated Col2a1 gene expression on days 3 (a), 7 (b), and 11 (c), and PTHrP overexpression inhibited BMP2-induced Col2a1 expression on days 3 and 7 (no statistical difference was found on day 11). **(B)** PTHrP overexpression inhibited BMP2-induced Col10a1 gene expression. BMP2 up-regulated Col10a1 gene expression on days 3 (a), 7 (b), and 11 (c), and PTHrP overexpression inhibited BMP2-induced Col10a1 expression on days 3, 7, and 11. **(C)** PTHrP overexpression promotes BMP2-induced aggrecan expression. Alcian blue staining was used to assess Aggrecan synthesis at days 7 and 11 (a); quantitative analysis also showed that PTHrP overexpression promoted BMP2-induced Aggrecan synthesis on day 7 (a), but no statistical significance was found on day 11 (b). **(D)** PTHrP overexpression inhibits BMP2-induced ALP activity. ALP staining (a) and ALP reading (b) were used to assess ALP activities at days 7 and 11 (a); quantitative analysis also showed that PTHrP overexpression inhibited BMP2-induced ALP activities on day 11, but no statistical significance was found on day 7 (b). ^##^*p* < 0.01, ^###^*p* < 0.001, and ^####^*p* < 0.0001, compared with the control group. ∗*p* < 0.5, ∗∗*p* < 0.01, ∗∗∗*p* < 0.001, and ∗∗∗∗*p* < 0.0001, compared with the indicated group.

### PTHrP potentiates BMP2-induced chondrogenic differentiation of MSCs and is essential for maintaining cartilage phenotype *in vivo*

To further investigate the regulatory role of PTHrP in BMP2-induced chondral–osteogenic differentiation of MSCs *in vivo*, we conducted subcutaneous MSC implantation studies of ectopic bone formation in athymic nude mice. Subconfluent MSCs were first transduced with AdGFP, AdBMP2, AdPTHrP, AdsimPTHrP, AdBMP2/AdPTHrP, or AdBMP2/AdsimPTHrP for 48 h, and were collected for subcutaneous injection. Ectopic masses were retrieved at 4 weeks and 8 weeks after implantation (Fig. 3A). Three-dimensional reconstruction images and transverse sections of microCT imaging data of the retrieved ectopic masses indicated that both PTHrP overexpression and silencing PTHrP expression led to decreased bone formation (Fig. 3B, a, b), while no detectable masses were retrieved from the AdGFP, AdPTHrP, or AdsimPTHrP groups. Quantitative analysis showed that PTHrP overexpression increased the total volume (TV) of the masses, compared with BMP2 alone, which was significantly nullified by silencing PTHrP expression at week 4 (Fig. 3C, a), although the potentiation effect of PTHrP on BMP2 was not significant at week 8 (Fig. 3D, a). Analyses of bone volume (BV) and the ratio of BV/TV showed that both PTHrP overexpression and silencing PTHrP decreased BV and BV/TV value at week 4 (Fig. 3C, b, c), while the differences were not apparent at week 8 (Fig. 3D, b, c), suggesting that PTHrP may play an important role in delaying BMP2-induced osteochondral differentiation of MSCs.

**Figure 3:**
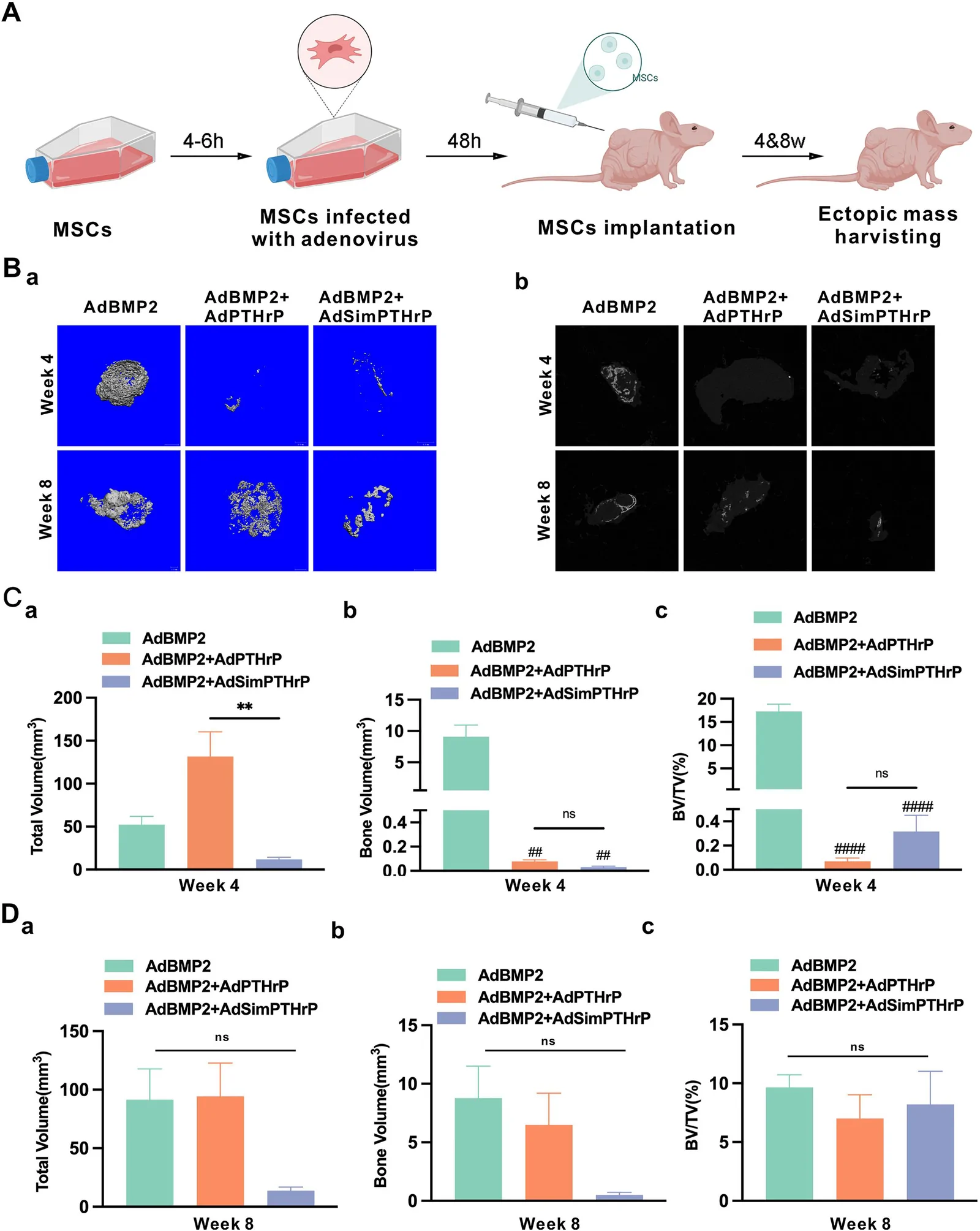
PTHrP inhibits BMP2-induced ectopic bone formation *in vivo*. **(A)** A diagram summarizes the process of subcutaneous mesenchymal stem cell (MSC) implantation. The image was drawn using Adobe Photoshop, contributed by Zhenglin Zhu. **(B)** Three-dimensional reconstruction (a) and transverse sectional (b) images for the representative ectopic masses. **(C)** Quantitative analysis of TV (a), BV (b), and the ratio of BV/TV for 4-week samples. **(D)** Quantitative analysis of TV (a), BV (b), and the ratio of BV/TV for 8-week samples. ^##^*p* < 0.01, ^###^*p* < 0.001, and ^####^*p* < 0.0001, compared with the control group. ∗*p* < 0.5, ∗∗*p* < 0.01, ∗∗∗*p* < 0.001, and ∗∗∗∗*p* < 0.0001, compared with the indicated groups. ns, no significance.

Histological evaluation (hematoxylin–eosin staining) and cartilage-specific staining (safranin O fast blue staining) revealed that BMP2 induced robust chondrogenic and osteogenic differentiation at week 4, in which both hypertrophic and trabecular bone were observed although most of the masses were in cartilage form, consisting with the fact that BMP2 induces endochondral ossification (Fig. 4A). At the same timepoint, PTHrP overexpression further suppressed BMP2-induced trabecular bone formation, leading to a predominant cartilage phenotype although silencing PTHrP diminished BMP2-induced chondrogenic differentiation and trabecular bone formation (Fig. 4A). At week 8, BMP2-induced chondrocytes were replaced by mature trabecular bone, although PTHrP overexpression still effectively suppressed BMP2-inudced endochondral ossification and retained predominantly cartilage phenotype (Fig. 4B), indicating an arrested hypertrophic–chondrogenic differentiation phenotype in the presence of exogenous PTPrP. Similar to the results observed in week 4, silencing PTHrP expression significantly diminished BMP2-induced endochondral differentiation and trabecular bone (Fig. 4B). Further quantitative analysis of cartilage matrix production revealed that PTHrP effectively enhanced BMP2-induced chondrogenic differentiation and cartilage matrix formation (Fig. 4C, a, b). Conversely, silencing PTHrP expression remarkably inhibited BMP2-induced chondrogenic and osteogenic differentiation of MSCs (Fig. 4C). The expression of chondrogenic marker Col2a1 and endochondral ossification marker Runx2 was also detected, which indicates that PTHrP promotes BMP2-induced chondrogenic differentiation and inhibits BMP2-induced endochondral ossification of MSCs *in vivo* (Fig. S2). The above *in vivo* findings strongly suggest that PTHrP may be an essential mediator for BMP2-induced endochondral differentiation and subsequent ossification of MSCs.

**Figure 4:**
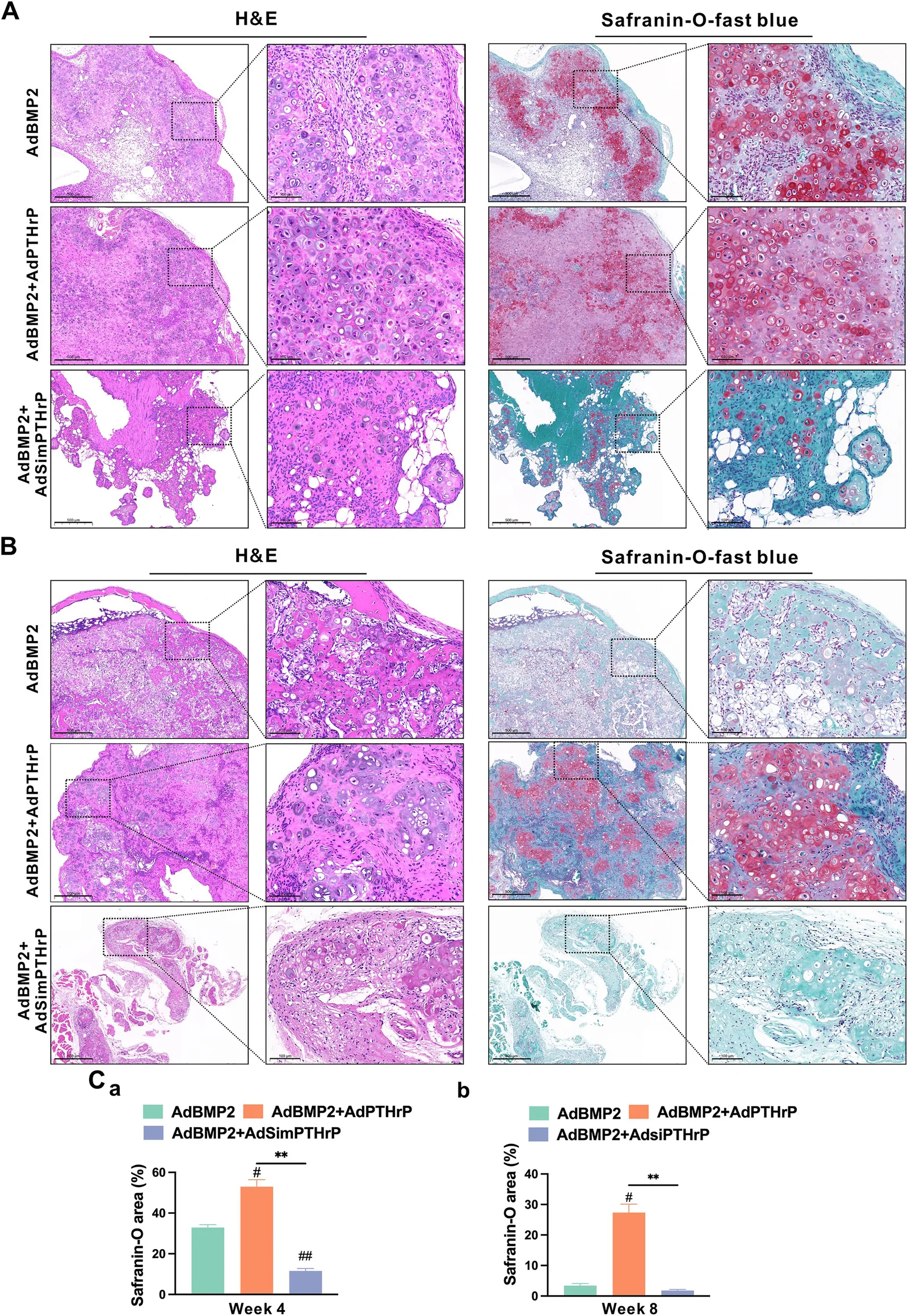
PTHrP promotes BMP2-induced chondrogenic differentiation of mesenchymal stem cells (MSCs) *in vivo*. **(A)** PTHrP potentiated BMP2-induced chondrogenic and inhibited hypertrophic differentiation of MSCs in 4-week samples. Hematoxylin–eosin staining (right two panels) and Safranin-O-fast blue staining (left two panels). **(B)** PTHrP potentiated BMP2-induced chondrogenic and inhibited hypertrophic differentiation of MSCs in 8-week samples. Hematoxylin–eosin staining (right two panels) and Safranin-O-fast blue staining (left two panels). **(C)** Quantitative analysis of Safranin-O areas in 4-week samples (a) and 8-week samples (b). ^##^*p* < 0.01, ^###^*p* < 0.001, and ^####^*p* < 0.0001, compared with the control group. ∗*p* < 0.5, ∗∗*p* < 0.01, ∗∗∗*p* < 0.001, and ∗∗∗∗*p* < 0.0001, compared with the indicated group. Scale bar: 500 μm in left panels and 100 μm in right panels.

### PTHrP mediates BMP2-induced hypertrophic–chondrogenic differentiation of MSCs through inactivating PI3K–AKT signaling

We further investigated the potential regulatory mechanisms through which PTHrP may mediate BMP2-induced hypertrophic chondrogenic differentiation of MSCs. We found that BMP2 stimulation induced the expression of the key chondrogenic transcription factor Sox9 and hypertrophic differentiation factor Runx2, which was effectively inhibited by silencing endogenous PTHrP expression at the protein level, confirmed by quantitative analysis (Fig. 5A, a–c). Conversely, PTHrP overexpression augmented BMP2-induced expression of Sox9, but much less pronounced on Runx2 expression, qualitatively and quantitatively (Fig. 5B, a–c). These results indicate that PTHrP may mediate BMP2-induced endochondral ossification upstream of Sox9, and to a much lesser extent, Runx2.

**Figure 5:**
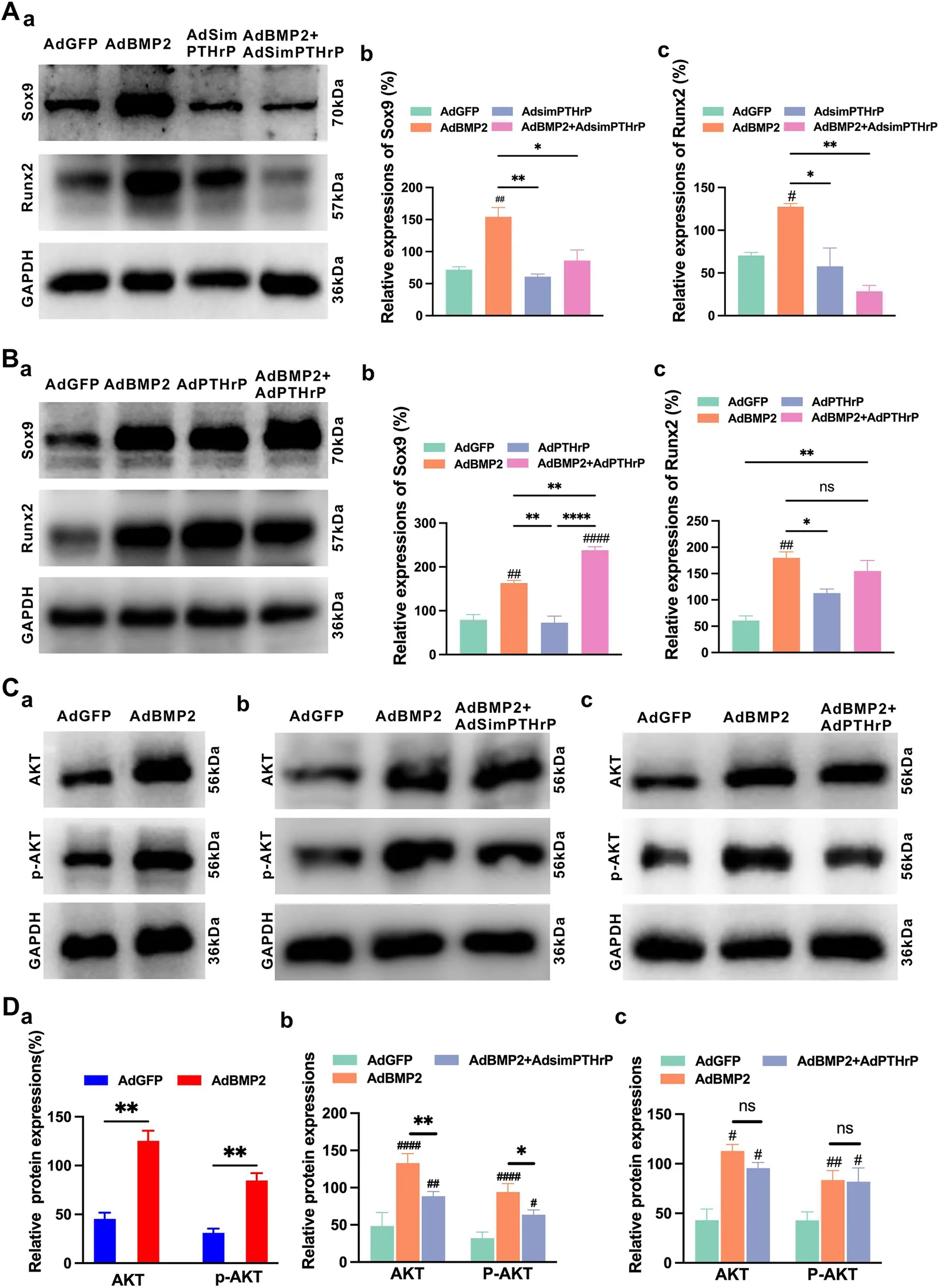
PTHrP potentiates BMP2-induced chondrogenic and hypertrophic differentiation of mesenchymal stem cells (MSCs) by suppressing PI3K–AKT signaling. **(A)** Silencing PTHrP inhibited BMP2-induced Sox9 and Runx2 expression. BMP2-induced expression of Sox9 and Runx2 was inhibited by silencing PTHrP (a), and quantitative analysis was shown (b). **(B)** Overexpression of PTHrP potentiated BMP2-induced Sox9 expression while not affecting Runx2 expression. Western blotting analysis showed that PTHrP overexpression potentiated BMP2-induced Sox9 expression rather than Runx2 (a), and quantitative analysis (b) showed the same trend. **(C)** BMP2 activated ATK signaling. With the stimulation of BMP2, total AKT and phosphorylated AKT (p-AKT) were up-regulated (a). Silencing PTHrP down-regulated BMP2-induced total AKT and p-AKT expressions (b), while PTHrP overexpression did not affect total AKT and p-AKT expression (c). **(D)** Quantitative analysis of total AKT and p-AKT expression in BMP2-induced MSC differentiation (a), and silencing PTHrP (b) and PTHrP overexpression (c) in BMP2-induced MSC differentiation. ^##^*p* < 0.01, ^###^*p* < 0.001, and ^####^*p* < 0.0001, compared with the control group. ∗*p* < 0.5, ∗∗*p* < 0.01, ∗∗∗*p* < 0.001, and ∗∗∗∗*p* < 0.0001, compared with the indicated group.

We previously reported that PI3K–AKT signaling plays an essential role in BMP2-mediated osteogenic differentiation of MSCs.^31^ Here, we investigated the functional relationship between PTHrP and PI3K–AKT signaling activation in the context of BMP2 stimulation. We found that BMP2 stimulation induced increased levels of total AKT and phosphorylated AKT (Fig. 5C, a), which were inhibited by silencing endogenous PTHrP expression (Fig. 5C, b). However, PTHrP overexpression did not affect the expression of total AKT and phosphorylated AKT (Fig. 5C, c). Quantitative analyses of the Western blotting results in Figure 5C confirmed the AKT expression changes (Fig. 5D). These findings demonstrate that PTHrP may play an essential role in BMP2-induced activation of PI3K–AKT signaling. Using the well-established PI3K inhibitor Wortmannin (WOR), we found that WOR down-regulated BMP2-induced expression of Runx2, p-AKT, PI3KP85, and p-PI3KP85(Fig. 6A, a), which was further confirmed by quantitative analysis (Fig. 6A, b). Taken together, PTHrP promoted BMP2-induced chondrogenic differentiation by potentiating Sox9 expression and regulating hypertrophic chondrocyte differentiation by inactivating PI3K–AKT signaling (Fig. 6B), indicating an essential role of PTHrP in maintaining the cartilage phenotype of BMP2-induced endochondral ossification of MSCs.

**Figure 6:**
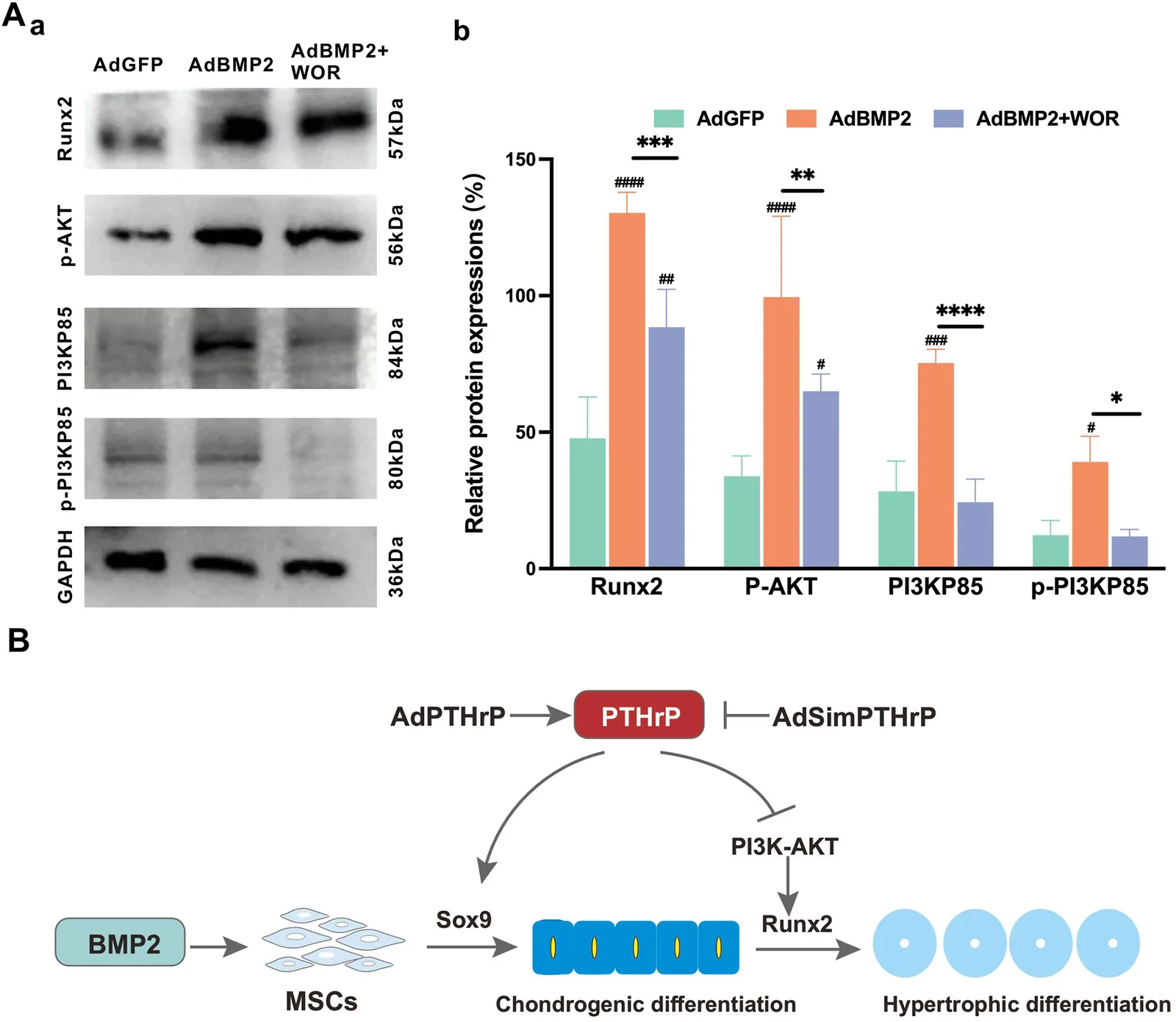
PI3K–AKT signaling mediates BMP2-induced hypertrophic differentiation that is inhibited by PTHrP. **(A)** PI3K–AKT inhibitor Wortmannin (Wor) suppresses BMP2-induced Runx2 expression and PI3K–AKT activation. WOR inhibited p-AKT, PI3KP85, and p-PI3KP85expression at the protein level (a), and quantitative analysis also showed these effects (b). ^##^*p* < 0.01, ^###^*p* < 0.001, and ^####^*p* < 0.0001, compared with the control group. ∗*p* < 0.5, ∗∗*p* < 0.01, ∗∗∗*p* < 0.001, and ∗∗∗∗*p* < 0.0001, compared with the indicated group. **(B)** PTHrP inhibited BMP2-induced hypertrophic differentiation of mesenchymal stem cells (MSCs) by activating PI3K–AKT signaling (graphical representation was contributed by Zhenglin Zhu on BioRender.com).

## Discussion

From degenerative diseases to traumatic injury, tissue engineering holds great potential for dealing with cartilage and bone pathologies.^36^, ^37^, ^38^, ^39^ BMP2 simultaneously stimulates osteogenic and chondrogenic differentiation, which has great potential for bone and cartilage tissue engineering.^14^^,^^29^^,^^31^^,^^34^ Hypertrophic–chondrogenic differentiation represents the transition phase between chondrogenic differentiation and endochondral ossification. Thus, it is important to investigate potential underlying mechanisms of BMP2-induced hypertrophic–chondrocyte differentiation of MSCs. In this study, we found that knockdown of PTHrP expression inhibited BMP2-induced chondrogenic, osteogenic, and hypertrophic differentiation. However, exogenous PTHrP effectively potentiated BMP2-induced Sox9 expression and chondrogenic differentiation with little effect on Runx2 expression. Mechanistically, PTHrP was shown to regulate hypertrophic–chondrogenic differentiation of BMP2-stimulated MSCs by inactivating PI3K–AKT signaling.

As one of the first identified BMPs, recombinant human BMP2 (rhBMP-2) has been approved by the US Food and Drug Administration for treating acute, open tibial shaft fractures and spinal fusion.^40^ Our previous work identified that BMP2-induced hypertrophic differentiation may be a major barrier for maintaining cartilage phenotype and BMP2-mediated cartilage tissue engineering.^14^^,^^29^^,^^41^ In this study, we found that BMP2-induced osteogenic and hypertrophic differentiation of MSCs was inhibited by PTHrP *in vivo*. Interestingly, silencing PTHrP blocked BMP2-induced both chondrogenic and osteogenic differentiation of MSCs. These results suggest the essential regulatory function of PTHrP in BMP2-induced osteogenic differentiation and chondrogenic differentiation of MSCs. On the other hand, PTHrP promoted Sox9 expression and inhibited Runx2 expression through PI3K–AKT signaling, suggesting that PTHrP may be essential for preserving cartilage phenotype.

PTHrP was first identified when studying hypercalcemia of malignancy, but the physiologic roles of PTHrP remain to be fully understood. In cartilage and endochondral bone formation, PTHrP plays important physiological roles. On the one hand, PTHrP regulates endochondral bone formation by strict coordination of temporally and spatially differentiation of chondrocytes to replace the cartilage template, which is cooperated with IHH signaling. On the other hand, PTHrP uses Gs activation of adenylyl cyclase/cAMP/protein kinase A pathway to keep chondrocyte proliferation.^21^^,^^22^^,^^41^ Simultaneously, PTHrP stimulates phosphorylation of the chondrocyte transcription factor Sox9, which may contribute to the action of PTHrP in restraining chondrocyte differentiation.^20^^,^^42^ Here, we found that overexpression of PTHrP inhibited the BMP2-induced hypertrophic differentiation of MSCs, suggesting the important role of PTHrP in maintaining cartilage phenotype upon BMP2 stimulation. Deng et al found that the regulation of PTHrP on chondrocytes was mediated by the activation of PI3K–AKT signaling.^43^ Simultaneously, Li et al reported that PI3K/Akt signaling exerted opposite effects on chondrocyte proliferation and hypertrophic differentiation of the condylar chondrocytes,^44^ which is consistent with our findings in BMP2-induced chondrogenic and hypertrophic differentiation of MSCs.

PTHrP also participates in osteogenic differentiation. Osteoblast-derived PTHrP was discovered in long bones and promoted bone defect repair by intramembranous bone formation.^23^ In addition, PTHrP deficiency resulted in inappropriate accumulations of glycogen, low bone mass, a decrease in trabecular thickness and connectivity, and an abnormally high number of adipocytes.^21^^,^^24^^,^^41^^,^^45^ Nevertheless, it has been noted that sustained high expression of PTHrP may lead to increased bone resorption and elevated bone turnover rates, thereby exerting negative effects on postnatal skeletal metabolism in the fetus.^46^ However, there is currently no direct evidence indicating that persistent PTHrP stimulation is the direct cause of typical postnatal bone loss, which requires further investigation.^46^, ^47^, ^48^, ^49^ Our study found that knockdown of PTHrP expression attenuated BMP2-induced osteogenic differentiation of MSCs, and overexpression of PTHrP inhibited BMP2-induced trabecular bone formation. These results indicated that PTHrP may inhibit hypertrophic differentiation of BMP2-induced endochondral ossification, which is essential for an effective cartilage and bone regeneration strategy. In terms of mechanism, we showed that, rather than regulating the expression of Runx2, PTHrP promoted chondrogenic differentiation of MSCs by PI3K–AKT signaling, which is consistent with our previous findings by transcriptomic landscape analysis.^31^ Nonetheless, it remains to be fully understood how PI3K–AKT signaling is inactivated, and whether crosstalk between PI3K–AKT and other signaling pathways, such as Smad, p38, and Notch signaling, exists. Ultimately, these lines of investigation should be beneficial for BMP2-based cartilage tissue regeneration. Furthermore, targeting PI3K–AKT signaling may hold the potential to assist BMP2-mediated cartilage regeneration.^50^^,^^51^ In summary, our study demonstrates that PTHrP plays a positive role in maintaining the chondrogenic phenotype and inhibiting hypertrophic differentiation in BMP2-induced MSC-based tissue-engineered cartilage by targeting the PI3K–AKT signaling pathway. This provides a new methodology and perspective for research on tissue-engineered cartilage construction and cartilage repair.

In conclusion, PTHrP promotes BMP2-induced chondrogenic differentiation while inhibiting BMP2-induced hypertrophic differentiation of MSCs by down-regulating PI3K–AKT signaling, indicating that PTHrP may play a potential role in BMP2-based cartilage tissue engineering.

## CRediT authorship contribution statement

**Shengqiang Gao:** Writing – original draft, Software, Methodology, Investigation, Formal analysis, Data curation. **Piao Zhao:** Writing – original draft, Methodology, Investigation, Data curation. **Tingting Du:** Writing – original draft, Methodology, Formal analysis, Data curation. **Yu Ye:** Software, Resources. **Shengwen Cheng:** Methodology, Investigation. **Senrui Liu:** Software, Formal analysis. **Chengcheng Du:** Visualization, Data curation. **Tong-Chuan He:** Writing – original draft, Project administration, Conceptualization. **Wei Huang:** Writing – review & editing, Supervision, Funding acquisition, Conceptualization. **Zhenglin Zhu:** Writing – review & editing, Supervision, Conceptualization. **Wei Xu:** Writing – review & editing, Validation, Methodology, Conceptualization. **Junyi Liao:** Writing – review & editing, Writing – original draft, Visualization, Validation, Supervision, Software, Resources, Methodology, Investigation, Funding acquisition, Formal analysis, Data curation, Conceptualization.

## Ethics declaration

The animal protocol of this study was reviewed and approved by the Ethical Committee of the First Affiliated Hospital of Chongqing Medical University (No. 2022-K92).

## Data availability

All data reported in this paper will be shared by the lead contact upon request.

## Funding

The reported work was supported by the 10.13039/501100001809National Natural Science Foundation of China (No. 81972069), Natural Science Foundation of Chongqing Science and Technology Commission (China) (No. CSTB2022NSCQ-MSX0814, CSTB2024NSCQ-KJFZZDX0024), Science and Technology Research Program of Chongqing Municipal Education Commission (China) (No. KJZD-K202500402), CQMU Program for Youth Innovation in Future Medicine (Chongqing, China) (No. W0154), and Innovation Project from Chongqing Municipal Education Commission (China) (No. CYB21169, CYS20192). J.Y. was supported by “Qiande” Talent Program–Outstanding Youth of The First Affiliated Hospital of Chongqing Medical University (Chongqing, China). Funding sources were not involved in the study design, in the collection, analysis, and interpretation of data; in the writing of the report; and in the decision to submit the paper for publication.

## Conflict of interests

T.C.H. is one of the editors-in-chief for *Genes & Diseases* and was not involved in the editorial review or the decision to publish this article. All authors declare that they have no known competing financial interests or personal relationships that could have appeared to influence the work reported in this paper. All other authors do not have any conflict of interests to declare.

## Figures and Tables

**figs1:**
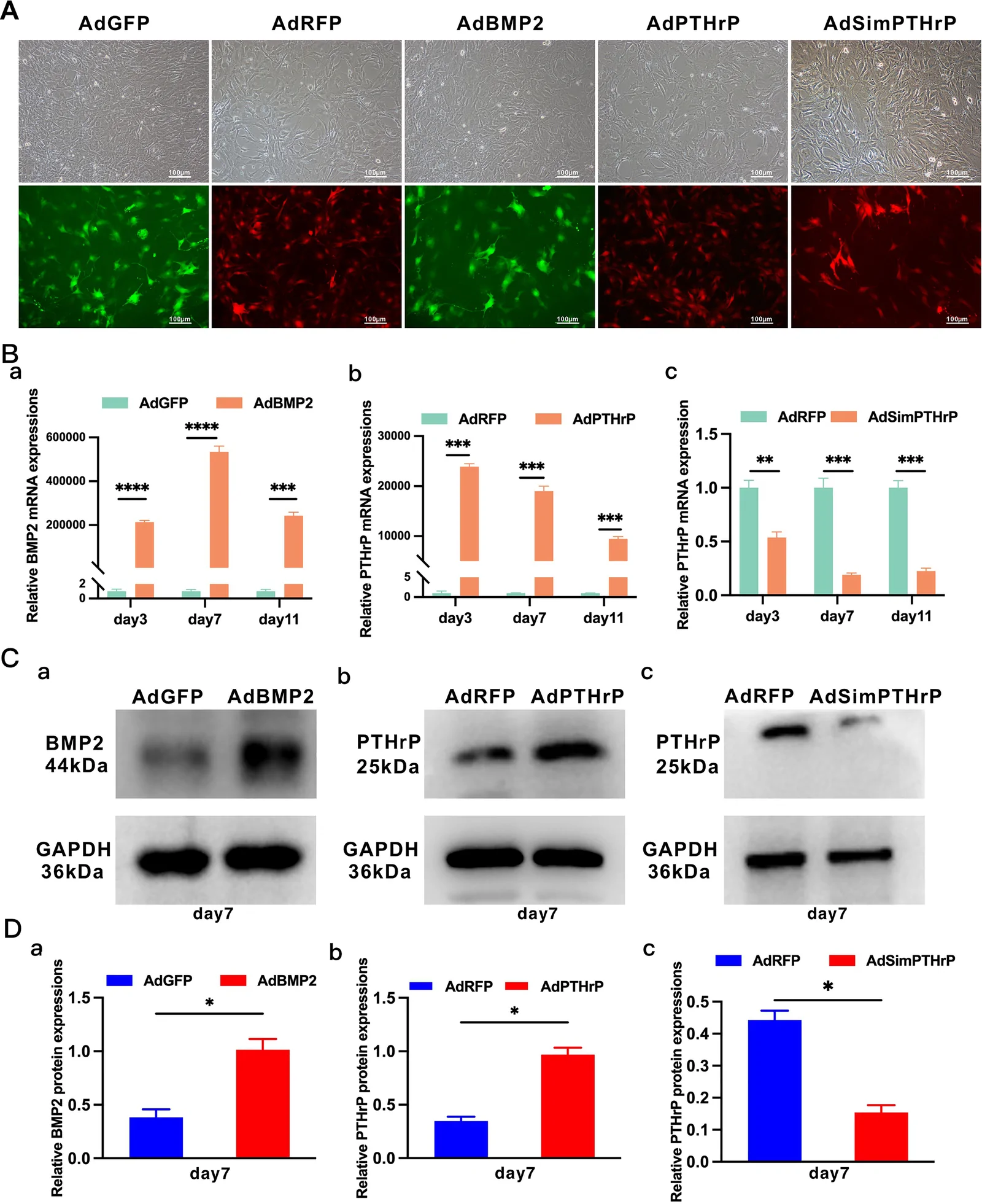


**figs2:**