# Supplementary material for: PTHrP suppresses BMP2-induced osteochondral differentiation and preserves cartilage phenotype in mesenchymal stem cells via inactivating PI3K–AKT signaling
Source: Genes Dis. 2026 Jun 5;14(2):102273. doi: 10.1016/j.gendis.2026.102273 (PMC13694792; doi:10.1016/j.gendis.2026.102273)
Supplement: Supplementary file 1 [file mmc1.docx]

**Figure S1** Adenovirus vectors effectively expressed PTHrP transgene and siRNAs against mouse PTHrP. **(A)** Adenovirus expression of GFP, RFP, BMP2, and PTHrP, and silencing PTHrP, effectively infected C3H10T1/2 cells. Bright light and fluorescent light images were presented. **(B)** Relative expression of target genes in different groups at different time points. AdBMP2-mediated overexpression of BMP2 (a), AdPTHrP-mediated overexpression of PTHrP, and AdSimPTHrP-mediated silencing of PTHrP (c) were presented. **(C)** Adenovirus-mediated target gene expression at the protein level. GAPDH was used as a housekeeping gene. **(D)** Quantitative analysis of target gene expression at the protein level. AdGFP and AdRFP were used as controls. ^##^*P* < 0.01, compared with the control group. ^*^*P* < 0.5, ^**^*P* < 0.01, ^***^*P* < 0.001, and ^****^*P* < 0.0001, compared with the indicated group.

**Figure S2** PTHrP promotes BMP2-induced chondrogenic differentiation and inhibits BMP2-induced endochondral ossification of mesenchymal stem cells (MSCs) *in vivo*. (A) PTHrP potentiated BMP2-induced Col2a1 expression of MSCs in 4- and 8-week samples (a). Quantitative analysis of positive areas was presented (b). **(B)** PTHrP potentiated BMP2-induced Runx2 expression of MSCs in 4- and 8-week samples (a). Quantitative analysis of positive areas was presented (b). ^##^*P* < 0.01 and ^###^*P* < 0.001, compared with the control group. ^**^*P* < 0.01 and ^****^*P* < 0.0001, compared with the indicated group. ns, no significance. Scale bar: 200 µm.

**Table S1** The primers for PCR analysis.

| Gene | Forward | Reverse |
| --- | --- | --- |
| *GAPDH (mouse)* | CTACACTGAGGACCAGGTTGTCT | TTGTCATACCAGGAAATGAGCTT |
| *Sox9 (mouse)* | AGGAAGTCGGTGAAGAACGG | GGACCCTGAGATTGCCCAGA |
| *Col2a1 (mouse)* | GCCAGGATGCCCGAAAATTAG | ACGATCACCTCTGGGTCCTT |
| *Runx2 (mouse)* | TGGCCGGGAATGATGAGAAC | AGGTGAAACTCTTGCCTCGT |
| *Col1a1 (mouse)* | GAGAGGTGAACAAGGTCCCG | AAACCTCTCTCGCCTCTTGC |
| *BMP2 (human)* | ACTCGAAATTCCCCGTGACC | CCACTTCCACCACGAATCCA |
| *Col10a1 (mouse)* | TCTCCCAGCACCAGAATCTAT | CTTTATGCCTGTGGGCGTTT |
| *PTHrP (mouse)* | ACTCCAAACCTGCTCCCAAC | GACCGAGTCCTTCGCTTCTT |
